# Novel antidotes for target specific oral anticoagulants

**DOI:** 10.1186/s40164-015-0020-3

**Published:** 2015-09-15

**Authors:** Arundhati Das, Delong Liu

**Affiliations:** Department of Medicine, New York Medical College and Westchester Medical Center, Valhalla, NY 10595 USA; Henan Cancer Hospital and the Affiliated Cancer Hospital of Zhengzhou University, Zhengzhou, China

## Abstract

Target specific oral anticoagulants (dabigatran, rivaroxaban, apixaban, and edoxaban) are changing the landscape of anticoagulation. The major drawback is the absence of an effective antidote for severe bleedings and/or prior to procedures. Currently there are a few promising antidotes undergoing clinical trials. This review summarized the latest development in idarucizumab, andexanet alpha and PER977.

## Background

Warfarin, a vitamin K antagonist (VKA), was the only oral anticoagulant for more than half a century [1]. There were various limitations like its narrow therapeutic index, food and drug interactions, need for regular monitoring and frequent dose adjustments. These drawbacks and limitations fuelled the development of newer agents [2]. In 2010, dabigatran, a direct thrombin inhibitor, became the first target specific anticoagulant approved by FDA [3]. It was soon followed by Factor Xa inhibitors-rivaroxaban and apixaban [4, 5]. Edoxaban has most recently joined the rest [6, 7].

They have been approved for prevention of stroke in nonvalvular atrial fibrillation, and recently in prevention of recurrent deep vein thrombosis and pulmonary embolism. These drugs are prescribed in fixed doses and have fewer incidents of intracranial hemorrhage in comparison to warfarin in large randomized phase III studies [8]. Nevertheless bleeding is still a relevant side effect and their biggest drawback has been the lack of a reliable reversible agent [9]. Unlike warfarin, there is no antidote for these newer agents. Currently there are a few promising antidotes undergoing clinical trials. These include idarucizumab, andexanet alpha and PER977 [10].

In this review we summarized studies on antidotes to the target specific oral anticoagulants, their mechanism of action and their potential in changing the future of anticoagulation.

### Target specific oral anticoagulants and the risk of bleeding

The development of TSOAs accelerated in the last decade. Various trials comparing warfarin to either dabigatran or any of the Factor Xa inhibitors proved that the newer agents had significantly lower bleeding risk [11, 12]. Ruff et al. did a meta-analysis comparing the safety and efficacy of the four newer oral agents to warfarin in patients with atrial fibrillation. They were found to be equally effective in the prevention of stroke. More importantly the incidence of intracranial hemorrhage was reduced by almost 50 % and there was a significant reduction in all cause mortality. However, an increase in gastrointestinal bleeding was observed [13]. Kham recently reported a case of spontaneous cardiac tamponade within 10 days of starting rivaroxaban on a patient [14]. Certain groups of patients on anticoagulation, such as the elderly with a fall, or those needing emergent surgeries or encountering trauma will need immediate reversal of anticoagulation [15]. This lack of antidote limits the use of TSOAs despite their many benefits.

At present, reversal of TSOAs is usually attempted by the administration of prothrombin complex concentrates (PCC). They contain Factor II, IX and X. The four Factor PCC also contains Factor VII. These agents are supposed to reverse the effect of the novel oral anticoagulants by saturating their action. However this rationale is yet to be proven by studies. Also this method does not neutralize the risk of thromboembolism [16].

### Dabigatran and idarucizumab

Dabigatran is a direct thrombin inhibitor administered as a low molecular weight prodrug dabigatran etexilate mesylate. After oral administration it converts to dabigatran, which is a reversible inhibitor of activated thrombin. With the RE-LY trial, dabigatran became the first FDA approved oral anticoagulant for the prevention of systemic thromboembolism and stroke in nonvalvular atrial fibrillation [17, 18]. Since then there have been noticeable incidents of bleeding. In a recent study comparing the risk of bleeding of dabigatran to warfarin, dabigatran was found to have an increased risk of major bleeding including gastrointestinal bleeds but a lower risk of intracranial hemorrhage [19]. Hemodialysis has been shown to clear 50–60 % of dabigatran and has been used to rapidly reduce massive bleeding [20], but standard hemodialysis is not a practical option in unstable conditions, where continuous venovenous hemodialysis has been successfully used [17, 21].

**Fig. 1:**
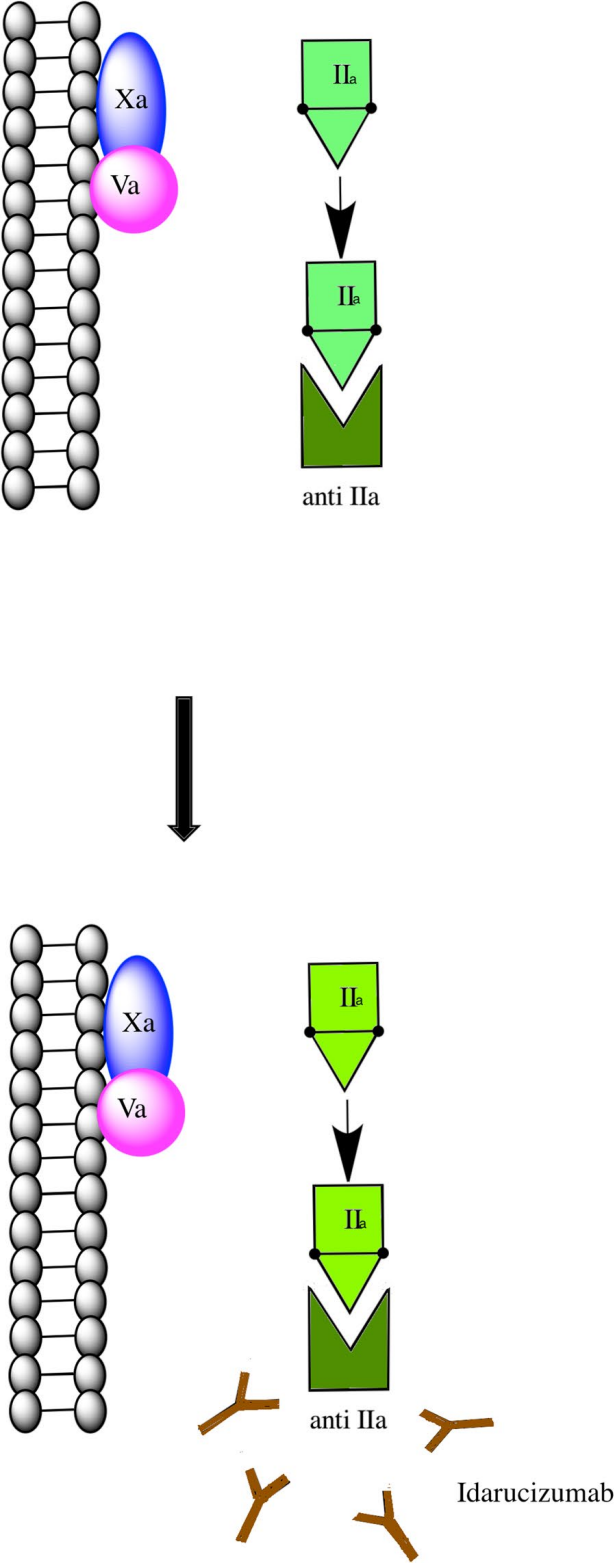
Idarucizumab binds and inactivates dabigatran. Idarucizumab is the first-in-class dabigatran-specific antidote. It is a humanized monoclonal antibody fragment [Fab] that binds specifically to and inactivates dabigatran

Idarucizumab (aDabi-Fab, BI 655075, UNII-97RWB5S1U6) is the first dabigatran specific antidote under study. It is a humanized monoclonal antibody fragment [Fab] that binds specifically to dabigatran (Fig. 1). It has an affinity for dabigatran that is ~350 times greater than that of thrombin. In ex vivo studies in rats, steady state dabigatran levels of ~200 ng were completely reversed within 1 min of an intravenous bolus of idarucizumab. Strong similarities were noted in the binding pattern of idarucizumab to dabigatran and thrombin to dabigatran. But idarucizumab lacks thrombin like enzymatic activity and does not bind thrombin substrates. Therefore it does not functionally resemble thrombin [22]. A phase 1, first-in-human, single-rising-dose, randomized, placebo-controlled trial in 110 healthy volunteers (27 placebo, 83 idarucizumab) was conducted to assess the pharmacokinetics, safety and tolerability of idarucizumab [23]. Idarucizumab was found to attain peak plasma levels rapidly. However its concentration decreased to 5 % or less of the peak level within 4 h secondary to renal elimination. It was found that it had to be dosed in 1:1 ratio with dabigatran for its complete efficacy. Also of importance is the fact that it had no impact on the coagulation profile of subjects who received placebo [23]. A phase 3 trial has been ongoing to evaluate the reversal of the anticoagulant effects of dabigatran by idarucizumab in patients treated with dabigatran who have uncontrolled bleeding or require emergency surgeries or procedures (REVERSE AD trial) [24–27]. An interim analysis showed that the anticoagulant effect of dabigatran was completely reversed within minutes by idarucizumab. This interim analysis included 90 patients. The primary end point of this study was the maximum percentage reversal of the anticoagulant effect of dabigatran within 4 h after the administration of 5 g of IV idarucizumab. This was determined by measuring the dilute thrombin time (elevated in 68 patients in the analysis) or ecarin clotting time (elevated in 81 patients) at a central laboratory. The median maximum percentage reversal was 100 % (95 % confidence interval, 100–100) in these patients with elevated coagulation profile [28].

### Factor Xa inhibitors and andexanet alfa

A recent analytical study was conducted in Germany using data from a prospective, noninterventional oral anticoagulation registry. 43 % of the 1776 patients on rivaroxaban reported bleeding and of those 6.1 % were classified as major bleeding [29].

**Fig. 2:**
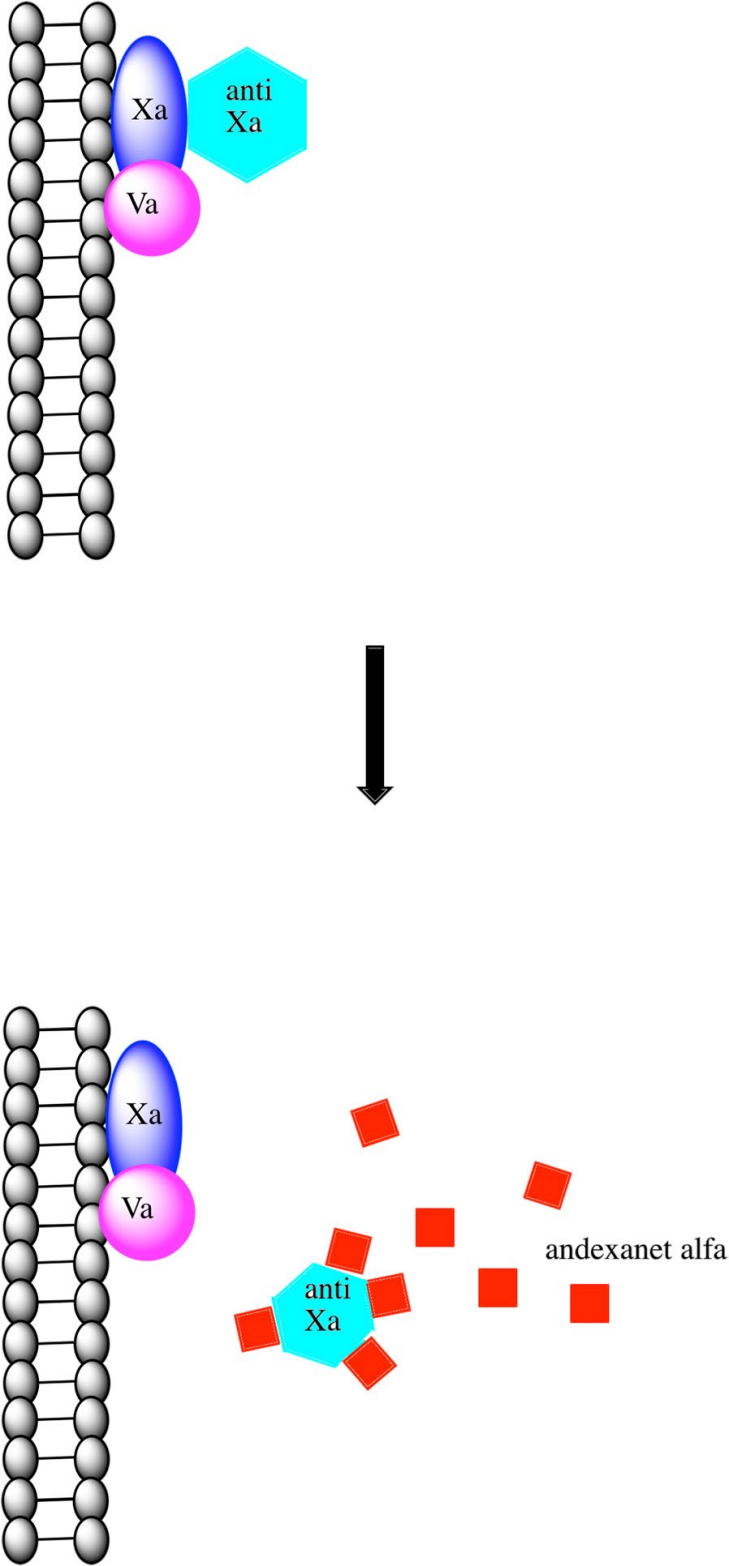
Andexanet alpha is a recombinant protein that binds to and inactivates direct factor Xa inhibitors. Andexanet lacks the membrane-binding γ-carboxyglutamic acid domain (Gla domain) of factor Xa, therefore lacks binding to the phospholipid membrane of cells

Andexanet alfa (PRT064445, r-Antidote; Portola Pharmaceuticals) is a 39 kDa, recombinant modified decoy of Factor Xa produced in Chinese hamster ovary cells. The amino acid serine is replaced by alanine at position 419. This recombinant protein retains the ability to bind direct FXa inhibitors as well as antithrombin activated by low molecular weight heparin or fondaparinux [30]. There was some concern that it could compete and hinder the function of Factor Xa in an active coagulation thus affecting hemostasis. Andexanet lacks the membrane-binding γ-carboxyglutamic acid domain (Gla domain) of Factor Xa which prevents its binding to the phospholipid membrane of cells (Fig. 2). This property thus allows the activation of Factor Xa by tissue factor and Factor VIIa via the extrinsic pathway [8, 30].

In a preclinical study in a rabbit model, andexanet alfa was found to reverse the action of direct Factor Xa inhibitors in a dose dependent manner [31]. The phase II studies with rivaroxaban and apixaban demonstrated a decrease in activity of Factor Xa by 53 and 20 % respectively. In a phase 2 randomized, double blind, placebo-controlled trial of andexanet, preliminary reports showed that a bolus dose of andexanet α antagonized the anti-Xa activity of apixaban and rivaroxaban (5 mg twice daily, 11 doses) in healthy subjects [32–35]. At present randomized placebo controlled double-blinded phase 3 trials are ongoing to evaluate the safety and efficacy of andexanet alfa in reversing apixaban- (ANNEXA-A study) and rivaroxaban-(ANNEXA-R study) induced anticoagulation in healthy volunteers.

### Target specific oral anticoagulants and PER977

PER977 (arapazine, ciraparantag; Perosphere Inc.) is a small, 512 Da, synthetic, water soluble molecule that binds to direct inhibitors of factor Xa and IIa as well as to heparin-based anticoagulants through non-covalent hydrogen bonding and charge interactions. It is reported to antagonize the effects of all anticoagulants except VKAs and agratroban [8, 30].

In an animal model, rats were overdosed with rivaroxaban, apixaban, edoxaban and dabigatran. PER977 was noted to reduce bleeding within 30 min of administration [36].

In the first human trial of PER977 (NCT01826266), the pharmacokinetics and pharmacodynamics were studied with escalating doses of PER977 (100–300 mg). It was tested on volunteers who were either untreated or pretreated with 60 mg of edoxaban. A dose of intravenous bolus of 300 mg PER977 was found to normalize whole blood clotting time within 10–30 min and the effect was sustained for over 24 h [37].

## Conclusion

Target specific oral anticoagulants represent a new era in anticoagulation. The major drawback is the absence of an effective antidote. With the ongoing clinical trials and preliminary results from the trials [28, 37], effective antidotes that can successfully reverse the effects of these novel anticoagulants may become available in the near future.
